# Age-dependent patterns of the gut microbiome, antibiotic resistome, and pathogenicity in captive koalas (*Phascolarctos cinereus*)

**DOI:** 10.1038/s42003-025-09302-2

**Published:** 2025-12-07

**Authors:** Hualong Su, Peiyun Han, Hongyu Yan, Chengcheng Wu, Shenzheng Zeng, Peng Zhang, Zhihui Wang, Jian Dong, Mincong Liang, Huang Jing, Danhua Zhang, Chen Yang, Naiyu Xie, Xinxin Liu, Shaoping Weng, Guixin Dong, Jianguo He

**Affiliations:** 1 Stat Key Laboratory of Biocontrol, Southern Marine Science and Engineering Guangdong Laboratory (Zhuhai), China-ASEAN Belt and Road Joint Laboratory on Mariculture Technology, Guangdong Provincial Key Laboratory of Aquatic Economic Animals, School of Life Sciences, Sun Yat-sen University, Guangzhou, China; 2 Institute of Ecology, College of Urban and Environmental Sciences, Peking University, Beijing, China; 3 School of Marine Sciences, Sun Yat-sen University, Zhuhai, China; 4 Chimelong Safari Park, Chimelong Group Co., Panyu, Guangzhou, China; 5 School of Environmental Science and Engineering, Sun Yat-sen University, Guangzhou, China; 6 State Key Laboratory of Marine Pollution (SKLMP), and Department of Chemistry, City University of Hong Kong, Hong Kong SAR, China

**Keywords:** Microbiome, Pathogens

## Abstract

Gut microbiome has a profound influence on koalas’ health. Yet, the relationships among the gut bacteriome, virome, antibiotic resistome, and pathogenicity throughout different stages in koala’s life remain elusive. Here, we presented a metagenome-resolved survey of gut microbiome utilizing 75 fecal samples from three groups of captive koalas. The diversity of bacteriome and virome were age-dependent, predominating in adult koalas. Lytic viruses increased with age as lysogenic viruses and bacterial hosts declined, and virus-to-microbe ratios rose, revealing concomitant age-related shifts in microbial communities, though causality remains unresolved. Antibiotic resistance genes (ARGs) were more prevalent in young koalas, unlike in humans, where they accumulate with age. Two ARG-carrying pathogens, *Klebsiella pneumoniae* and *Escherichia coli*, were identified and cultured, with *K. pneumoniae* and *E. coli* predominating in young koalas. One age-dependent lytic virus infecting *K. pneumoniae* only detected in young koalas, and two lysogenic viruses infecting *E. coli* identified the in young and adult koalas. Analyses showed a positive correlation between mobile genetic elements (MGEs) and virulence factors (VFs), which facilitated the widespread dissemination of VFs and impacted health. Collectively, this study advances the understanding of gut microbiome in health, providing solutions to the treatment and management of captive koalas.

## Abstract (web summary)

Age-dependent dynamics of gut bacteriome, virome, and antibiotic resistome reveal microbial and pathogenic shifts shaping health in captive koalas.

## Introduction

The koala (*Phascolarctos cinereus*), an arboreal marsupial species endemic to Australia, solely feeds on *Eucalyptus* leaves^1^. There are multiple threats that makes it an endangered species, such as a loss of habitat^2^, various diseases including diarrhea illnesses^3,4^, chlamydiosis^5^, and koala retrovirus (KoRV)^6^, etc. Recently, empirical studies have recognized gastrointestinal disorders as a significant contributor to the mortality^7–9^, shedding light on the essential role of the gut microbiome in host physiology^10,11^, encompassing aspects such as energy metabolism^12,13^, and host immune response^14,15^. Gut microbiome (pathogens and non-pathogens) of koalas are largely influenced by the age of captive koalas (*P. cinereus*), which is thought to play an essential role in health and disease^16^.

In the gut microbiota of koalas, *Streptococcus bovis* and *Lonepinella koalarum*, which have the potential to degrade lignin and tannin, showed age-dependent variations in their abundance profiles^17,18^. The bacterial pathogen *Klebsiella pneumoniae* has been reported to predominantly infects infant koalas^19^, with age being a significant factor in susceptibility. *K. pneumoniae* was found at very high abundance before and after mortality events in koalas^19^, implicating it as a potential contributor to disease and death. A previous report had associated *K. pneumoniae* with gut microbiome dysbiosis in koalas, with pouch young being particularly susceptible^20^. These infections may be treatable with lytic phage therapy, as shown in previous mouse model experiments^21^. Studies have explored the age-dependent patterns of gut phages diversity in the gut of human^22,23^, macaques^24^, chimpanzees^25^, and pigs^26^, etc. The diversity of human gut phages is influenced by age, increasing from childhood to adulthood and declining after age 65 in healthy populations^22^. As a major component of the gut microbial ecosystem, viruses (mainly phages) have been proved to have the potential to impact prokaryotic host dynamics via lytic infection^27,28^ and the animals’ health status^29,30^. In the gut environment, phages serve as essential regulators of bacterial host dynamics. Analyzing the virus-to-microbe abundance ratio (VMR) in the captive koalas’ gut is significant as it provides insights into both koalas’ health and ecological dynamics, serving as a valuable indicator of virus-host interactions. Although the diverse virus-host interactions dynamics in gut ecosystems have been explored^22,31–33^, the age-dependent patterns of these interactions across the entire lifespan still require further investigation.

The increasing prevalence of antibiotic resistance genes (ARGs) is recognized as a significant threat to public health, representing a global environmental pollutant^34–36^. Recently, antimicrobial resistance (AMR) has been observed in bacteria and viruses inhabiting the gut of mammals such as human^37^, pigs^38^, giant pandas^39^, monkeys^40^, gibbons^41^, and honeybees^42,43^, etc. ARGs whose hosts belongs to the bacteria and phages have been extensively explored to understand their diversity and abundance within the human microbiome^44^. The drastic rise in the incidence of ARGs-carrying pathogens (APs) is a major health problem, as these pathogens are common natural inhabitants of both human and animal microbiomes^45^. ARGs, mobile genetic elements (MGEs), and virulence factors (VFs) form a network that enhances pathogen antibiotic resistance and virulence, complicating treatment and posing public health challenges by promoting rapid pathogen adaptation in human and animal microbiomes^46,47^. Previous metagenomic studies of the koala gut have highlighted the critical role of gut bacteria in host physiology^16,48–50^. However, age-related dynamics of the gut microbiome, resistome, and pathogenicity in captive individuals remain largely unexplored.

Hence, in this study, we adopted a metagenomic approach to investigate the gut bacteriome and virome in captive koalas, categorized into three age-dependent groups. In addition, the composition and diversity of ARGs, ARG-harboring microbiome, pathogens, and their infecting viruses were also explored. To this end, metagenomic sequencing was performed on 75 fecal samples to characterize the koala gut microbiome, assess pathogenicity profiles, and explore the diversity of ARGs. Our analyzes contribute to advancing the understanding of the age-dependent patterns of gut bacteriome and virome ecosystem and provide practical insights into the risk assessment and treatment of captive koalas.

## Results

### Diverse and individualized Metagenome-assembled genomes in koala gut microbiome

**Fig. 1:**
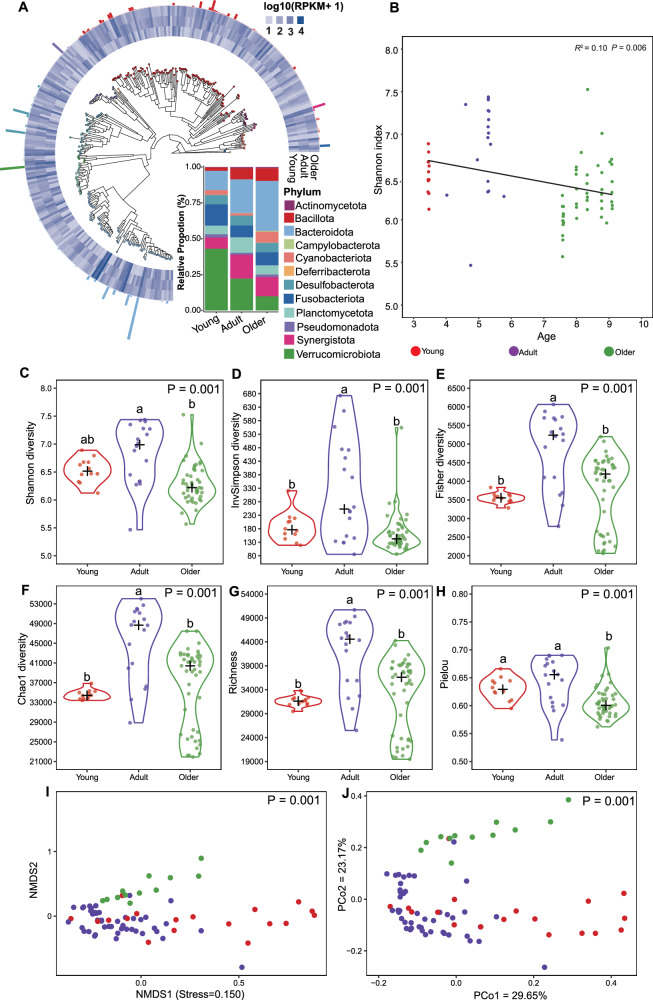
Composition, distribution, and diversity of koala gut bacteriome among three groups. **A** Phylogeny and taxonomic distribution of gut bacteriome among three groups. **B** Correlation between age and the Shannon index (*n* = 75 samples). The color spectrum represents three age-dependent groups, with first-degree polynomial fits shown in grey and black. **C**–**H** MAGs-based microbial Shannon, Invsimpsons, Fisher, Chao1, Richness, and Pielou diversity analysis in the three age-dependent groups (*n* = 75 samples). Statistical significance was evaluated via one-way ANOVA, with least significant difference (LSD) post-hoc analysis. Different lowercase letters indicate significant differences at *α* = 0.05. **I**, **J** Non-metric multidimensional scaling (NMDS) analysis and principal coordinates analysis (PCoA) based on the Bray–Curtis distance in the three age-dependent groups (*n* = 75 samples).

288 Metagenome-assembled genomes (MAGs) were identified from 75 fecal samples of captive koalas. Taxonomical annotation of MAGs was performed utilizing the Genome Database Toolkit (GTDB-Tk, “classify_wf”). These MAGs were classified into 50 bacterial families belonging to 12 phyla (Fig. 1A). For the abundance of bacterial lineages in three groups (young, adult, and older), Verrucomicrobiota (9.83%~43.00%) and Bacteroidota (13.46%~34.72%) were the two most dominant lineages, followed by Bacillota (2.27%~8.88%) and Fusobacteriota (8.41%~14.85%) (Fig. 1A). The genome annotations of 288 MAGs were shown (Supplementary Data 1). For the alpha diversity, the Shannon index was negatively correlated with age, showing a gradual decrease as koala aged (*R*^2^ = 0.11, *P* = 0.016) (Fig. 1B). The Invsimpsons, Fisher, Chao1, and Richness indices were significantly higher in the adult group compared to the young and older groups (*P* < 0.001) (Fig. 1D–G). The Pielou index was significantly higher in both the adult and older groups than in the young group (*P* < 0.001) (Fig. 1H), and the Shannon index was significantly higher in adult group compared to the older groups (*P* < 0.001) (Fig. 1C). Nonmetric multidimensional scaling (NMDS) and principal coordinates analysis (PCoA), based on Bray–Curtis dissimilarity, showed significantly distinct mirusviral community structures among three groups (PERMANNOVA; *R*^2^ = 0.28, *P* < 0.001) (Fig. 1I, J). These findings uncovered that koala gut microbial diversity is significantly influenced by age group differences and other unresolved ecological factors.

### Diverse and previously uncharacterized viral genomes associated with age in the koala’s intestine

**Fig. 2:**
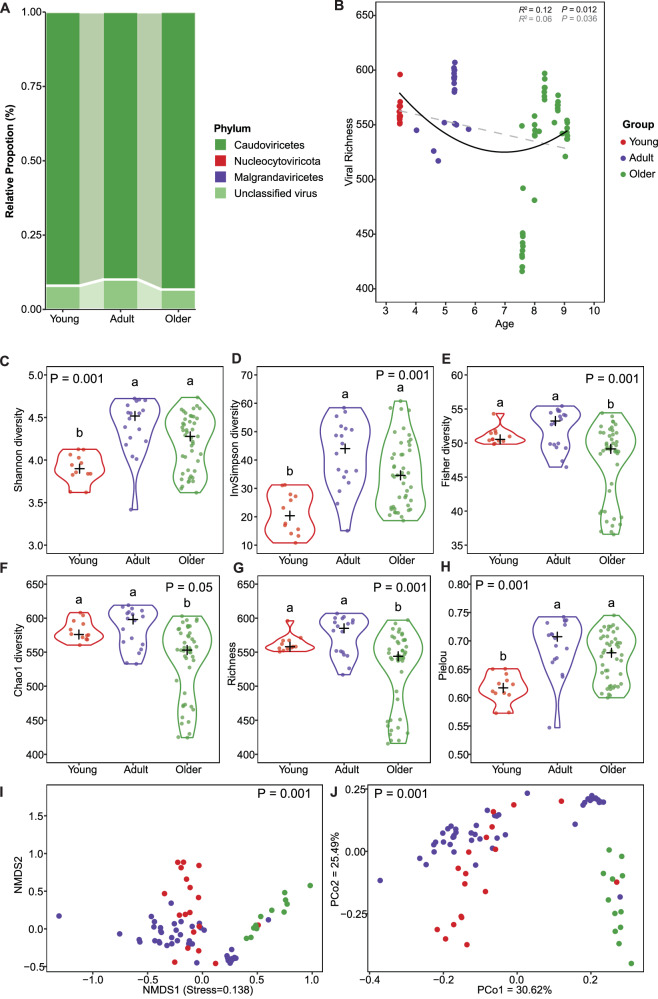
Composition, distribution, and diversity of koala gut virome among three groups. **A** Taxonomic distribution of gut virome among four groups. **B** Correlation between age and the viral Richness (*n* = 75 samples). The color spectrum represents four age-dependent groups, with first- and second- degree polynomial fits shown in grey and black, respectively. **C**–**H** vOTUs-based microbial Shannon, Invsimpsons, Fisher, Chao1, Richness, and Pielou diversity analysis in the three age-dependent groups (*n* = 75 samples). Statistical significance was evaluated via one-way ANOVA, with least significant difference (LSD) post-hoc analysis. Different lowercase letters indicate significant differences at *α* = 0.05. **I**, **J** Non-metric multidimensional scaling (NMDS) analysis and principal coordinates analysis (PCoA) based on the Bray–Curtis distance in the three age-dependent groups (*n* = 75 samples).

Assembled contigs were employed to manually identified the putative viral sequence using the mentioned workflows. A total of 671 representative viral operational units (vOTUs) (1.0 kb~1352.238 kb) were obtained. Viral richness was negatively correlated with age (*R*^2^ = 0.12, *P* = 0.012), indicating a gradual decline in overall richness during ageing (Fig. 2B). Multiple alpha diversity indices, including Shannon, Invsimpsons, and Pielou were significantly lower in the young group compared with adults and olders (*P* < 0.001) (Fig. 2C, D, H). Fisher, Chao1, and Richness indices were notably higher in young and adult groups compared to the older group (*P* < 0.05) (Fig. 2E–G). Beta diversity analyzes further revealed distinct clustering of samples by age group, as shown by NMDS and PCoA ordinations (PERMANNOVA; *R*^2^ = 0.29, *P* < 0.001), highlighting age-dependent shifts in koala gut virome composition and other unresolved ecological factors (Fig. 2I, J). In the taxonomic classification and abundance analysis of viral lineages across three groups, *Caudoviricetes* (89.94%~93.26%) comprised the most dominant lineage, and *Malgrandaviricetes* was detected at a relatively low abundance (Fig. 2A). Remarkably, taxonomic classification of the vOTUs showed that the vast majority (77.31%) correspond to previously uncharacterized viruses across multiple taxonomic ranks. The conserved TerL protein was used for the phylogenetic analysis of the class *Caudoviricetes*. In the phylogenetic tree of the class *Caudoviricetes*, a total of 18 unique vOTUs from the family *Autographiviridae* (*n* = 16), *Casjensviridae* (*n* = 1), and *Herelleviridae* (*n* = 1) were widely distributed in the tree (Fig. S1). These 18 distinct viral genome sequences exhibited significant phylogenetic distances from the known RefSeq genomes. Notably, these sequences, positioned on separate branches, suggesting they may represent previously uncharacterized viruses.

### Age-dependent patterns of gut lytic and lysogenic virus-host interactions in captive koalas

73 MAGs were identified as hosts of lytic and lysogenic viruses, and the number of lytic and lysogenic viruses were 223, and 166, respectively. As age increased, the abundance of lytic viruses significantly rose, while lysogenic viruses and bacterial hosts showed a notable decline (Fig. S2A). In addition, the VMR steadily increased from youth to old age, possibly reflecting an enhanced viral replication cycle with age (Fig. S2B). Together, these findings demonstrate that bacterial and viral communities displayed concomitant age-related dynamics, although the directionality of these associations remains unresolved.

### Overview of the ARG profiles and diversity in the koala’s intestine

**Fig. 3:**
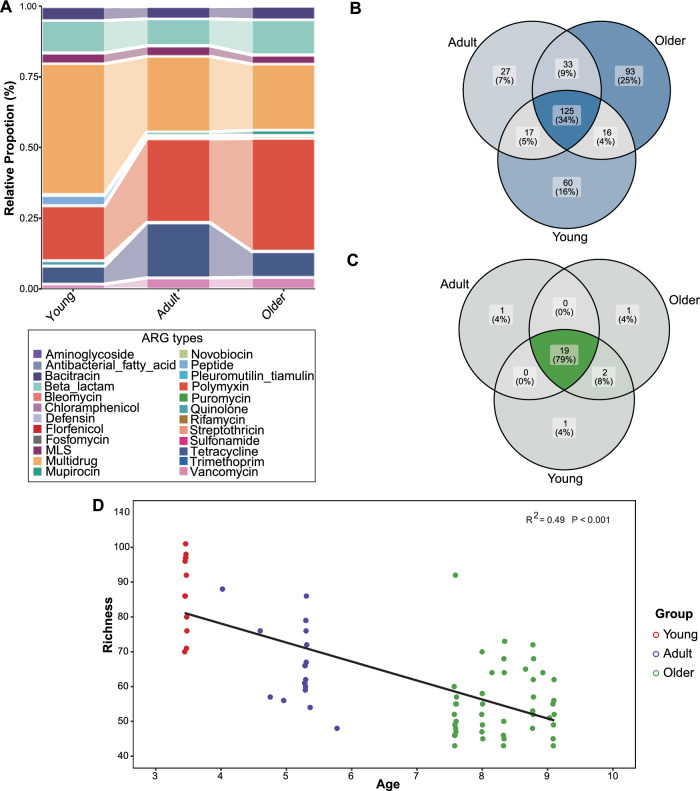
Annotation overview of gut microbiome ARGs among three groups. **A** The composition and abundance of ARG types in koalas’ gut microbiome. **B** Comparisons among three groups shared ARG subtypes. **C** Comparisons among three groups shared ARG types. **D** Correlation between age and the amount of ARG subtypes richness. Linear regression, *R*^*2*^ and *P* value are displayed (*n* = 75 samples).

High throughput sequencing reads were assigned to detect the distribution and abundance of ARGs in samples. A total of 24 ARG types and 617 ARG subtypes were identified in the fecal samples (Fig. 3A). Interestingly, ARG richness was negatively correlated with age (*R*^2^ = 0.49, *P* < 0.001), indicating a gradual decline in overall richness during ageing (Fig. 3D).

**Fig. 4:**
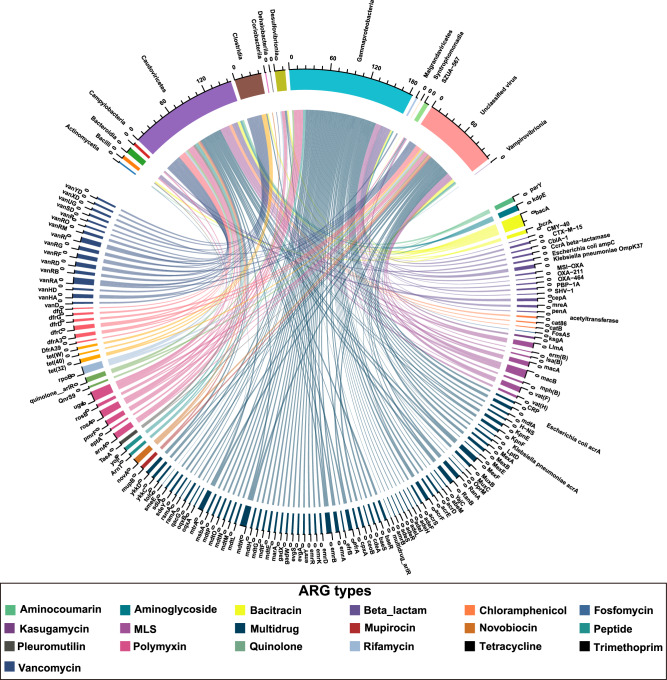
The association between ARGs (at the subclass level) and their potential hosts, including bacteria and viruses.

In terms of the abundance of the most prevalent ARG types across the four groups, multidrug resistance (30.96% and 38.49%) predominated in the young and adult groups, respectively, while polymyxin resistance (42.36%) was most abundant in the older groups (Fig. 4). Polymyxin resistance (25.90% and 23.42%) ranked second in abundance in the young and adult groups, respectively, whereas multidrug resistance (18.43%) was the second most abundant in the older group. Tetracycline resistance (18.56% and 11.54%) ranked third in abundance in the young and adult groups, while beta-lactam resistance (11.05%) held the third position in the older group. Beta-lactam resistance (6.23% and 10.70%) ranked fourth in abundance in the young and adult groups, while tetracycline resistance (10.36%) held the fourth position in the older group. Additionally, there are some unique types and subtypes of ARG presenting in the three groups (Fig. 3B, C). Distinct ARG types were exclusively associated with specific age groups: Bleomycin in the young, Streptothricin in adults, and Puromycin in olders (Fig. 3C). The number of unique ARG subtypes in the young, adult, and older groups were 60, 27, and 93, respectively (Fig. 3B).

### Overview of ARG putative hosts in bacterial and viral communities

To explore the hosts of ARGs, we identified and selected ARG-carrying contigs (ACCs) from MAGs and vOTUs sequences using reference database SARG v3.0. A total of 465 ACCs included 265 ACCs from bacterial contigs and 200 ACCs from viral contigs. In the bacterial group ACCs, the most abundant bacterial lineages were Gammaproteobacteria (54.11%), Bacteroidia (20.49%), Clostridia (19.19%), and Desulfovibrionia (2.61%), respectively (Fig. 4). Meanwhile, in the viral group ACCs, *Caudoviricetes* was the dominant viral lineage, making up 67.91% of the total. Additionally, a significant portion of the viruses, accounting for 32.09%, were identified as unclassified within the ACCs (Fig. 4). The main antibiotic classes associated with Gammaproteobacteria hosts primarily included multidrug (67.37%), polymyxin (10.44%), and beta-lactam (6.65%). Meanwhile, Bacteroidia-ACCs contained predominantly beta-lactam (99.08%) and a small percentage of polymyxin (0.92%). For Clostridia-ACCs, the dominant antibiotic classes were vancomycin (65.49%) and tetracycline (29.89%). Additionally, in the viral group ACCs, *Caudoviricetes* hosts were primarily resistant to polymyxin (51.49%), macrolide-lincosamide-streptogramin (MLS, 16.73%), and multidrug (8.65%). For unclassified viruses within the ACCs, the most abundant known antibiotic classes were multidrug (38.65%), MLS (21.73%), and vancomycin (8.86%) (Fig. 4).

### Diversity and composition of and ARG-carrying pathogens and their infecting viruses

**Fig. 5:**
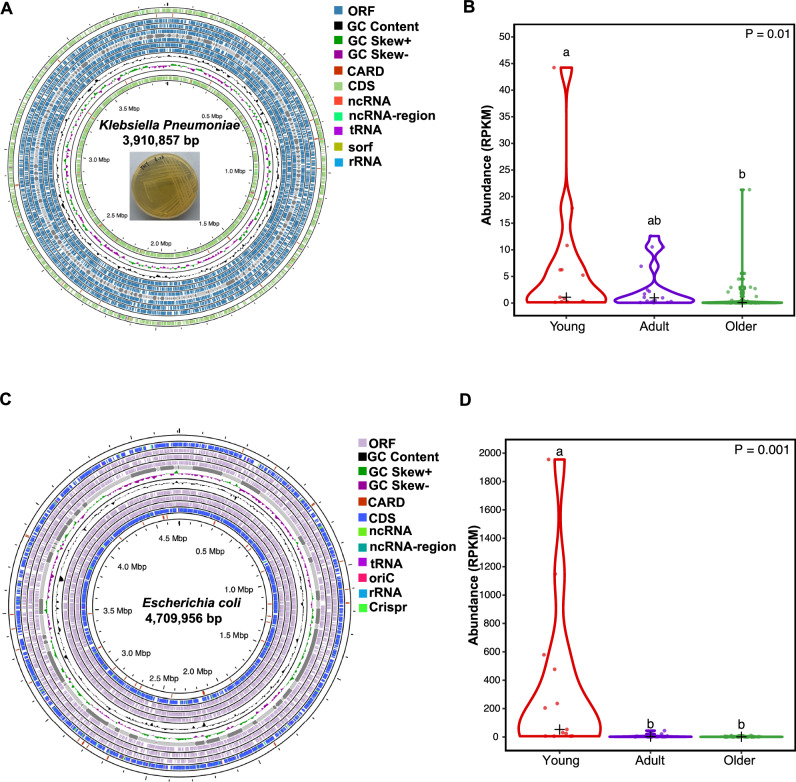
Genomes annotation and distribution of two ARG-carrying pathogens (*Klebsiella pneumoniae* and *Escherichia coli).* **A** The genome-wide annotation of *Klebsiella pneumoniae*. **B** Comparison of the abundance of *Klebsiella pneumoniae* among three groups. Statistical significance was evaluated via one-way ANOVA, with least significant difference (LSD) post-hoc analysis. Different lowercase letters indicate significant differences at *α* = 0.05. **C** The genome-wide annotation of *Escherichia coli*. **D** Comparison of the abundance of *Escherichia coli* among three groups. Statistical significance was evaluated via one-way ANOVA, with least significant difference (LSD) post-hoc analysis. Different lowercase letters indicate significant differences at *α* = 0.05.

To identify gut microbial pathogens in koala fecal samples, taxonomic annotation of metagenomic sequences was conducted to the reference pathogen database. Based on the identification of gut pathogens in koala samples to explore age-dependent patterns, only two pathogens were detected in our study: *K. pneumoniae* and *Escherichia coli*. This analysis revealed the presence of two ARG-carrying pathogens: *K. pneumoniae* (Fig. 5A) and *E. coli* (Fig. 5C). The *K. pneumoniae* genome had a completeness of 100% with a contamination of 1.08%, whereas the *E. coli* genome showed a completeness of 98.4% and contamination of 0.16%. To investigate the distribution of these two ARGs-carrying pathogens among the three age-dependent groups, we calculated their abundance. The abundance of *K. pneumoniae* in the young group was notably higher than the older groups (*P* < 0.01) (Fig. 5B). For the *E. coli*, the abundance in the young group was significantly higher than in the other two groups (*P* < 0.001) (Fig. 5D).

**Fig. 6:**
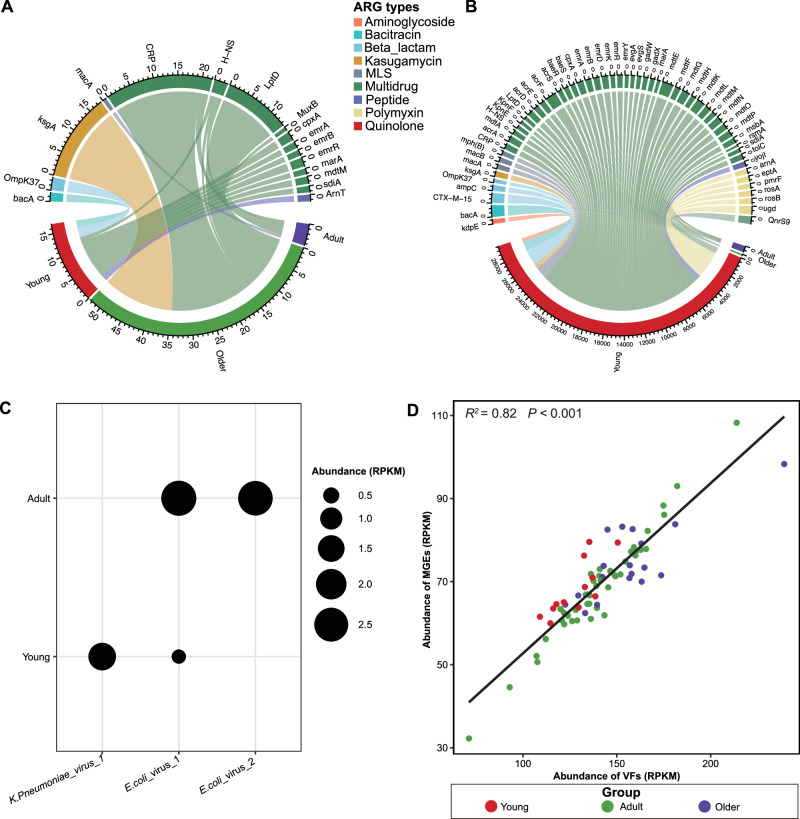
An overview of the antibiotic resistome in two pathogens (*Klebsiella pneumoniae* and *Escherichia coli*), and the distribution of their infecting viruses. **A** Antibiotic resistome analysis of *Klebsiella pneumoniae* (at subclass level). **B** Antibiotic resistome analysis of *Escherichia coli* (at subclass level). **C** Distribution of three viruses infecting these two pathogens among young and older groups (lytic *K. pneumoniae*_virus_1 infecting *Klebsiella pneumoniae* only detected in young koalas, and the other two lysogenic viruses infecting *Escherichia coli* identified in young and adult koalas). **D** Correlation between the abundance of VFs and MGEs. Linear regression, *R*^*2*^ and *P* value are displayed (*n* = 75 samples).

*K. pneumoniae* and *E. coli* were predicted to exhibit resistance to various types of antibiotics, including multidrug, beta-lactam, polymyxin, MLS, peptides, aminoglycoside, bacitracin, and kasugamycin (Fig. 6A). Additionally, *E. coli* was predicted to be resistant to Quinolone (Fig. 6B). Additionally, we identified that koala gut symbionts, such as *L. koalarum*, a bacterium previously recognized as a key gut symbiont, harbored ARGs and MGEs in their genomes.

To investigate the potential of viruses capable of infecting these two APs, several approaches were employed to predict viral hosts. Surprisingly, a total of three viral genomes were predicted to infect these two APs, including one virus infecting *K. pneumoniae* and two viruses infecting *E. coli*. The viral genome, dubbed *K. pneumoniae*_virus_1 and predicted to infect *K. pneumoniae*, was detected exclusively in the young group. (Fig. 6C). It belonged to the family *Casjensviridae*, had a genome length of 58,303 bp with 99.21% completeness and no host contamination, and was annotated as a lytic virus. For the other two genomes, which were predicted to infect *E. coli*, were detected in young and adult samples (Fig. 6C). One of them belonged to the order of *Caudoviricetes*, the other was annotated as unclassified virus, dubbed *E coli*_virus_1, and *E coli*_virus_2, respectively. The genomes of *E coli_*virus_1 and *E coli*_virus_2 had the length of 312,936, and 13,123 bp, respectively, having a quality genome with 56.90%, and 100% completeness, respectively. They were annotated as lysogenic viruses.

### The interactive association among ARGs, MGEs, and VFs

We explored the correlation between the abundance of MGEs (Supplementary Data 2) and VFs (Supplementary Data 3). Notably, Statistical analysis showed a significantly positive linear relationship between the abundance of MGEs, and VFs described by the model (*R*^2^ = 0.82, *P* < 0.001) in Fig. 6D.

## Discussion

As an endangered mammal, the koala stands as a symbol of wildlife conservation, with the age-dependent patterns of its gut bacteriome and virome remaining a topic of interest. Here, we characterized the gut microbiome, antibiotic resistome and pathogenicity of captive koalas, dissecting their age-dependent patterns and identifying their unique microbial signatures. We found that the diversity and abundance of bacterial, viral, ARGs, and pathogens were age-dependent and diverse community with exclusive microbial populations across the three groups.

We retrieved a multitude of 288 MAGs and 671 vOTUs. The diversity and abundance of prokaryotes were highest in the adult group, lower in the young and older groups. This may be related to their diet and developmental stage, as young koalas, which primarily feed on their mother’s fecal pap^51^ (a unique substance with higher microbial density and rare taxa abundance than typical feces) before transitioning to eucalyptus leaves, have a less developed microbiome compared to adults, limiting their ability to break down and detoxify the toxic compounds in eucalyptus^52^. These findings correspond to the patterns observed in humans, where microbial diversity is initially low in infants, then peaks in adults, and then gradually declines with age^53–55^.

Consistent with recent human and primate evidence, gut microbiome diversity exhibits marked age-related remodeling. In humans, infancy is characterized by rapid gains in alpha diversity, followed by relative stability in adulthood and compositional divergence in older age, where “microbiome uniqueness” increases and associates with survival outcomes^56,57^. Parallel patterns in nonhuman primates show that while alpha diversity may not uniformly decline in late life, communities become less stable and more individualized with age^58,59^. In koalas, age is likewise a key determinant of gut community structure, against a backdrop of strong individual signatures and maternal/foraging influences on alpha diversity^60–62^. Together, these studies reinforce that age-related differences in diversity (and stability/individualization) are biologically conserved across mammals, providing a robust comparative context for our findings.

Similarly, we found that adult koalas exhibited notably higher^59^ diversity and abundance compared to young and older koalas. This age-related variation may be due to several factors, including the environmental exposure^63^ and immunosenescence^64,65^ in the gut ecosystem. As they transition from juveniles to adults, their digestive and immune systems mature, creating a more stable habitat for diverse viral communities^22^.

Recent studies demonstrate that gut viral diversity is strongly age-dependent. In infants, viromes are typically dominated by lytic phages and exhibit high variability but relatively low overall diversity. With maturation, viral communities expand in richness and become more stable, often enriched in lysogenic phages^66^. In older individuals, however, several cohorts report a decline in viral diversity accompanied by increased inter-individual heterogeneity, suggesting reduced stability of the gut virome in late life^67,68^. These observations indicate that virome diversity follows a trajectory of early expansion, midlife stabilization, and late-life contraction, broadly paralleling—but not always mirroring—age-related bacterial dynamics.

Additionally, our findings reveal coordinated but contrasting trajectories of bacterial hosts and viruses across koala age groups. The decline of bacterial hosts alongside the increase in lytic viruses and virus-to-microbe ratios suggests that age is accompanied by shifts in microbial community balance^69^, potentially reflecting changes in host physiology or immune regulation that alter the virus–bacteria relationship^70^. The reduced prevalence of lysogenic viruses with age may indicate a diminished role for lysogeny in older individuals, while the relative rise of lytic viruses points to more active viral replication in aging microbiomes^71^. However, whether these changes are driven by viral dynamics acting on bacterial hosts, by bacterial community decline facilitating viral expansion, or by shared external factors remains unresolved. Future longitudinal and experimental studies will be critical to disentangle these relationships and to clarify how age shapes the balance between bacterial and viral components of the koala gut microbiome.

A total of 24 ARG types and 617 ARG subtypes were identified in the koala fecal samples. ARG richness declined with age, suggesting a contraction of the koala gut resistome over time. This contrasts with earlier research on human gut microbiota, which showed that ARG richness tends to accumulate with age, with the highest levels found in older individuals^72^. This pattern may partly reflect higher antibiotic exposure in juveniles^19,73,74^, who are more susceptible to infections, leading to elevated ARG diversity early in life that diminishes with reduced treatment in adulthood. In addition, age-related loss of bacterial diversity, together with shifts in diet, immune function, and environmental exposures, could further contribute to the observed decline^72^. These findings highlight ageing and life history as important factors shaping resistance gene diversity in wildlife microbiomes.

Subsequently, our findings revealed that Gammaproteobacteria-ACCs were the dominant bacterial lineage, and *Caudoviricetes*-ACCs were the dominant viral lineage. The findings are consistent with previous studies showing that Gammaproteobacteria is the most abundant class of bacteria harboring ARGs^75^ and represents a significant portion of the gut microbiome in various animals, including migratory birds^76^, dogs^77^, and pigs^78^. Additionally, *Caudoviricetes*, a dominant class within virus-ACCs, has been widely found across diverse animal species^79^ and environment^80^.

The positive linear model demonstrated a strong positive correlation between the abundance of MGEs and VFs. For some APs, MGEs are a major mechanism for acquiring VFs^81–83^. In diarrheagenic *E. coli* (EHEC), MGEs are crucial for defining pathotypes by facilitating the acquisition of virulence factors, exemplified by the presence of Shiga toxin in EHEC^84–86^. Considering these previous findings, our results imply that MGEs may facilitated the moderate dissemination of VFs within the gut microbiome of koalas, contributing to the potential spread of pathogenic traits. It highlights the need for further investigation into the role of MGEs in promoting virulence within wildlife microbiomes and its broader implications for host health and disease dynamics. The finding that *K. pneumoniae* and *E. coli* were all more abundant in the young koalas highlights age-related differences in the gut microbiota of captive koalas.

*K. pneumoniae*, a member of the Enterobacteriaceae family, is a well-known Gram-negative pathogen associated with numerous infections in both humans and animals^87,88^. Its presence in younger koalas is particularly concerning, as it is a major cause of neonatal sepsis^89,90^, a condition that poses significant risks to the survival of juvenile koalas^91,92^. The higher abundance of *K. pneumoniae* in young koalas could be due to their underdeveloped immune systems, which may be less capable of controlling opportunistic pathogens^20^. The presence of *K. pneumoniae* in older koalas was also detected in a previous investigation^93^, which could be linked to age-related immune decline, making them more susceptible to infections. Given that *E. coli* often serves as an early colonizer in the mammalian gut, its higher prevalence in juveniles may reflect an immature and less complex microbial community structure at this life stage^94^. Frequent medical interventions, including antibiotic treatments, may further favor the expansion of opportunistic taxa such as *E. coli* in young animals^95^. By contrast, the reduced abundance of *E. coli* in adults may indicate the establishment of a more stable and competitive gut ecosystem, where commensal bacteria can limit its dominance^96^. These results underscore the role of age in shaping gut microbial composition and suggest that management practices for captive koalas should account for the heightened vulnerability of juveniles to shifts in gut microbiota.

Furthermore, these two pathogens were predicted to exhibit resistance to various types of antibiotics, making it particularly challenging to treat related disease outbreaks in koalas with antibiotic therapies. In addition, previous studies reported that gut symbionts exchanged ARGs with pathogens via horizontal gene transfer, facilitated by MGEs^97^. In our study, we detected the presence of ARGs and MGEs in koala gut symbionts, including *L. koalarum*, which is a key cellulose-degrading bacterium in the koala gut^98^. Our findings indicate that koala gut symbionts could potentially exchange ARGs with pathogens. While experimental validation is still needed, the co-occurrence of ARGs and MGEs emphasizes the importance of investigating gene exchange and its role in AMR dissemination. Overall, these findings indicate that antibiotic-resistant pathogens are age-dependent, highlighting the need for enhancing screening and monitoring procedures to reduce koala mortality in the future.

Surprisingly, three viruses predicted to infect these two bacterial pathogens were identified, and their genomes were characterized. The *K. pneumoniae*_virus_1, a lytic phage from the family *Casjensviridae* that infects *K. pneumoniae*, was detected exclusively in the young group, while the other two lysogenic viruses infecting *E. coli* were detected in the young and adult groups. In cases of severe infections resistant to multiple antibiotics, lytic phage therapy is gaining recognition as a promising alternative treatment^99^. Existing studies have demonstrated the efficacy of lytic phages in treating various *K. pneumoniae*-induced infections, including bone^100^, pulmonary^101^, and urinary tract infections^102^. Given that *K. pneumoniae*-related diseases are a leading cause of mortality in juvenile koalas^19^, the application of lytic phage therapy presents a previously uncharacterized and potentially effective strategy for combating pathogenic bacterial infections in captive koalas. This approach could address the growing challenge of antibiotic resistance, offering a targeted, sustainable solution for managing bacterial outbreaks in vulnerable wildlife populations. Further research into the safety, efficacy, and delivery of phage therapy in koalas is warranted to optimize its use in conservation and rehabilitation efforts. In summary, these findings illuminate age-dependent patterns in the gut microbiome and their associations with the antibiotic resistome and pathogenicity, providing practical insights for koala conservation and clinical management.

## Methods

### Sample collection, DNA extraction, metagenomic sequencing

The captive koalas at Guangzhou Chimelong Safari Park in China were fed a standardized diet of fresh eucalyptus leaves, primarily *Eucalyptus robusta* and *Eucalyptus tereticornis*. All koalas included in this study were born and raised in captivity at the zoo. They are descendants of 13 founder individuals originally imported as captive animals from the Currumbin Wildlife Sanctuary, Australia. Since birth, these koalas have lived exclusively in the zoo environment, with no history of exposure to natural habitats or the wild. We have complied with all relevant ethical regulations for animal use. A total of 75 fecal samples were collected from these captive koalas, which were then divided into four groups based on their age: young (1 to 3 years old, *n* = 12), adult (4–6 years old, *n* = 18), and older (7–9 years old, *n* = 45), corresponding to juvenile, fully mature adult, and later adult stages, respectively^60,103^. Note that only a single fecal sample was collected from each animal, representing a snapshot of the gut microbiome at the time of sampling.

The fecal samples were transported at 4 °C and subsequently stored at −80 °C in the laboratory until DNA extraction was conducted. The QIAamp PowerFecal DNA Kit (QIAGEN, USA) was used to extract the total DNA according to the manufacturer’s directions. For metagenomic sequencing, to prepare DNA fragments for Illumina sequencing with PCR amplification, DNA fragments were sonicated to 2*150 bp, then end-polished and ligated with the full-length adaptor. Sequencing libraries were prepared using the NEXTFLEX Rapid DNA-Seq Kit (Cat. No. NOVA-5144, PerkinElmer, USA) according to the manufacturer’s instructions. Libraries were sequenced on the Illumina NovaSeq 6000 platform using the NovaSeq 6000 S4 Reagent Kit v1.5 (300 cycles, Cat. No. 20028312, Illumina, USA).

### Metagenome assembly and binning

The raw reads were filtered using fastp^104^ with the following parameters: (-q 20 -u 30 -w 12 -y -l 50 -r -W 5 -M 20) for generating the clean pair-end reads. MEGAHIT (Version 1.1.3)^105^ was employed to assemble the clean reads into contigs with default parameters. MAGs were generated from assembled contigs using the binning module of MetaWRAP v1.3.2^106^ (with maxbin2, concoct, and metabat2), followed by the bin_refinement module (parameters: -c 50 -x 10). After dereplication using dRep v3.1.1 with parameters (-comp 50 -con 10 -sa 0.95)^107^, a non-redundant set of 288 species-level MAGs was obtained. All 288 representative MAGs were taxonomically assigned using the classify_wf workflow of GTDB-Tk^108^ (v1.6.0) with default settings, based on the Genome Taxonomy Database (GTDB, release 202).

### Virus identification, taxonomic assignment, and host prediction

Virus identification pipelines refer to a previous study^109^ based on the following rules: (1) contigs (≥1 kb) with high or medium-quality genome completeness, or containing terminal repeats, as annotated by CheckV v1.0.1^110^, were automatically selected. (2) contigs (≥10 kb) needed to have virus score above 0.8 and either contain at least one virus hallmark, identified by geNomad^111,112^, or had a virus marker score of at least 5.0. (3) Contigs ranging from 5 to 10 kb were required to have a geNomad virus score above 0.9, include at least one identified virus hallmark, and show virus marker enrichment higher than 2.0. Viral genomes were clustered into 671 representative species-level vOTUs using CD-HIT v4.8.1^113^ (95% sequence identity and 85% alignment coverage). The geNomad v1.3.3 (end-to-end mode)^111,112^ was used for taxonomic assignment of viral genomes, adhering to the taxonomy outlined in ICTV’s VMR number 19 (https://ictv.global/). Taxonomic classification was further refined to the subfamily level used vConTACT3 v3.1.3 (https://bitbucket.org/MAVERICLab/vcontact3/src/master/).

Multiple host prediction strategies were used to associate viral genomes with their microbial hosts, utilizing the approach described in previous studies^114,115^. (1) CRISPR spacer matches were identified by predicting CRISPR arrays in koala gut microbial genomes using CRISPRidentify v1.1.0^116^ with default parameters. Spacers (<25 bp) and CRISPR array containing fewer than three spacers were excluded. CRISPR spacers were aligned with viral genomes utilizing BLASTn, allowing ≤1 mismatch, and employing a chosen threshold of 95% identity, 95% coverage and *E*-value of 1e-5. (2) tRNAscan-SE v2.0.9^117^ (-B -A mode) was used to identify the shared tRNA between virus and their host. In addition, sequences were aligned using Blastn with parameters: ≥90 identity and a query coverage of ≥ 95%. (3) For alignment-based strategy, viral genomes were aligned with microbial genomes using BLASTn, considering their nucleotide sequence homology with the following criteria: *E*-value  ≥  0.001, nucleotide identity ≥70%, match coverage over the length of viral genomes ≥75%, and bitscore ≥50. (4) WIsH^66^, RaFAH^118^, VirHostMatcher (VHM)^119^, and Prokaryotic virus Host Predictor^120^ were separately performed utilizing iPHoP v1.1.0^121^. The microbial genome was predicted as the potential viral host whose taxonomy belonged to the same family with top hits based on multiple above methods. The lifestyles of vOTUs, categorized as temperate or lytic, were predicted using PhaTYP^122^ and VIBRANT^123^. Based on the previous studies^23,33^, the VMR was calculated as:

$${{{\rm{VMR}}}}=\frac{{\sum }_{n}{Abundance\; of}{vOTUs\; targeting\; bacterial\; genu}{s}_{i}}{{Abundance\; of\; bacterial\; genu}{s}_{i}}$$

### ARGs identify and calculate abundance at reads levels

Clean reads were aligned to the reference database SARG v3.0 to acquire the annotation of ARGs profiles utilizing ARG-OAP v3.2.4 with default parameters^124–126^. A total of 24 ARGs types and 617 ARGs subtypes were identified in 75 fecal samples. The ARGs abundance was normalized by ppm (reads per one million reads).

### Identification of ARGs host in prokaryotic and viral sequences

Metagenomic sequences were employed to detect open reading frames (ORFs) using Prodigal v2.6.3^127^. The prokaryotic amino acid sequences were aligned using Blastp against the non-redundant SARG v3.0 database^126^ with specific parameters: *E*-value threshold of ≤ 10^−10^, a similarity of ≥70%, and a query coverage of ≥70%. The identification of prokaryotic genomes harboring ARGs was conducted following approaches described in previous studies^39,128,129^. In addition, viral amino acid sequences were aligned as queries in Blastp against the non-redundant SARG v3.0 database with specific parameters: *E*-value threshold of ≤10^−5^ and a query coverage of ≥80%. The approach of identification ARGs host in viral sequences refers to several previous studies^80,130,131^.

### Identification of pathogen, mobile genetic elements, and virulence factor

The approach to select the reference pathogen list refers to a prior study^132^. A pathogenic database containing 876 pathogenic species was constructed. The taxonomic assignment of metagenomic contigs to pathogens was based on the taxonomic output generated by GTDB-Tk v1.4.1^108^ and geNomad v1.3.3^111,112^. All ORFs protein sequences were aligned using BLASTP against MGEs database^133^ with specific parameters: ≥80% amino acid identity, and a query coverage of ≥70%^134^. Another approach to identify the MGEs was utilizing the non-redundant annotation result of kofamscan^135^ by keywords summarized from a prior study^136^. PlasFlow v1.1^137^ was employed with default parameters to predict plasmid and chromosomal sequences. The amonic acid sequences of ACCs were employed using diamond Blastp against virulence factor (VF) database (http://www.mgc.ac.cn/VFs/) with *E*-value threshold of ≤10^−7^ ^138^. We employed eggnog-mapper^139^ v2.1.12 with database^140^ v5.0.2 and METABOLIC^141^ v4.0 to perform functional annotation of MAGs. By integrating functional annotation results with evidence from existing literature, we identified MAGs exhibiting gut symbiotic functions and showing no known pathogenic features as candidate gut symbionts.

### Phylogenetic analysis and abundance profiles

The MAGs amino acid sequences were aligned with MAFFT v7.310^142^. Subsequently, alignments were trimmed using trimal v1.4.1^143^ with the ‘automated1’ option. Model selection and phylogenetic tree construction were conducted using IQ-TREE2 (v2.1.4-beta)^144^ under the specified parameters: (-s -st AA -alrt 1000 -bb 1000 -ntmax 36 -mem 180 G -T AUTO). The tree was visualized utilizing iTOL v6^145^. For the 18 previously uncharacterized viruses which belonged to *Caudoviricetes*, a proteomic tree was constructed via VipTree server^146^. To estimate the abundance of MAGs, vOTUs, pathogens, ARGs, MGEs, and VFs, clean paired reads were mapped to contigs or genes utilizing bowtie2 v2.3.3^147^ with default parameters. SAMtools was used to transfer sam files into bam files. The RPKM (Reads per kilobase per million mapped reads) values were used to represent the abundance of MAGs and vOTUs. CoverM (filter and contig modes) (parameters: --min-read-percent-identity 95 --min-read-aligned-percent 75 -m rpkm --trim-min 10 --trim-max 90 --min-covered-fraction 70) were utilized to calculate RPKM-normalized abundances of contigs or genes across samples^132,148,149^.

### Statistics and reproducibility

Alpha diversity metrics were computed using the “microeco” package^150^ (v1.14.0). Phylogenetic diversity, Bray–Curtis-based beta diversity, NMDS, and PCoA were generated with the vegan package^151^ (v2.6-4) and visualized using ggplot2 (v3.5.2) in R (v4.5.0) within the RStudio Server environment (v2025.05.0.496). Statistical significance was considered at *p* ≤ 0.05. Sample sizes are indicated and described in the corresponding figure legends.

### Reporting summary

Further information on research design is available in the Nature Portfolio Reporting Summary linked to this article.

## Supplementary information

Supplementary Information

Description of Additional Supplementary Files

Supplementary Data 1

Supplementary Data 2

Supplementary Data 3

Reporting Summary

## Data Availability

All other data necessary for the conclusions are included in this manuscript. The complete sequencing data are publicly accessible at the China National Center for Bioinformation (https://ngdc.cncb.ac.cn) under the accession number PRJCA036472. All raw datasets used to generate the figures in this manuscript are available through Figshare at the following DOI: 10.6084/m9.figshare.30618116.v2.

## Code availability

The complete original code, as well as the datasets used for figure generation, have been deposited on GitHub and are publicly available. Workflows and supporting scripts can be accessed at: https://github.com/HualongSu/Koala-gut-bacteriome-and-virome-project.
