# Supplementary material for: Age-dependent patterns of the gut microbiome, antibiotic resistome, and pathogenicity in captive koalas (Phascolarctos cinereus)
Source: Commun Biol. 2025 Dec 7;9:40. doi: 10.1038/s42003-025-09302-2 (PMC12783762; doi:10.1038/s42003-025-09302-2)
Supplement: Supplementary file 1 — Supplementary Information [file 42003_2025_9302_MOESM1_ESM.pdf]

Supplementary Figures

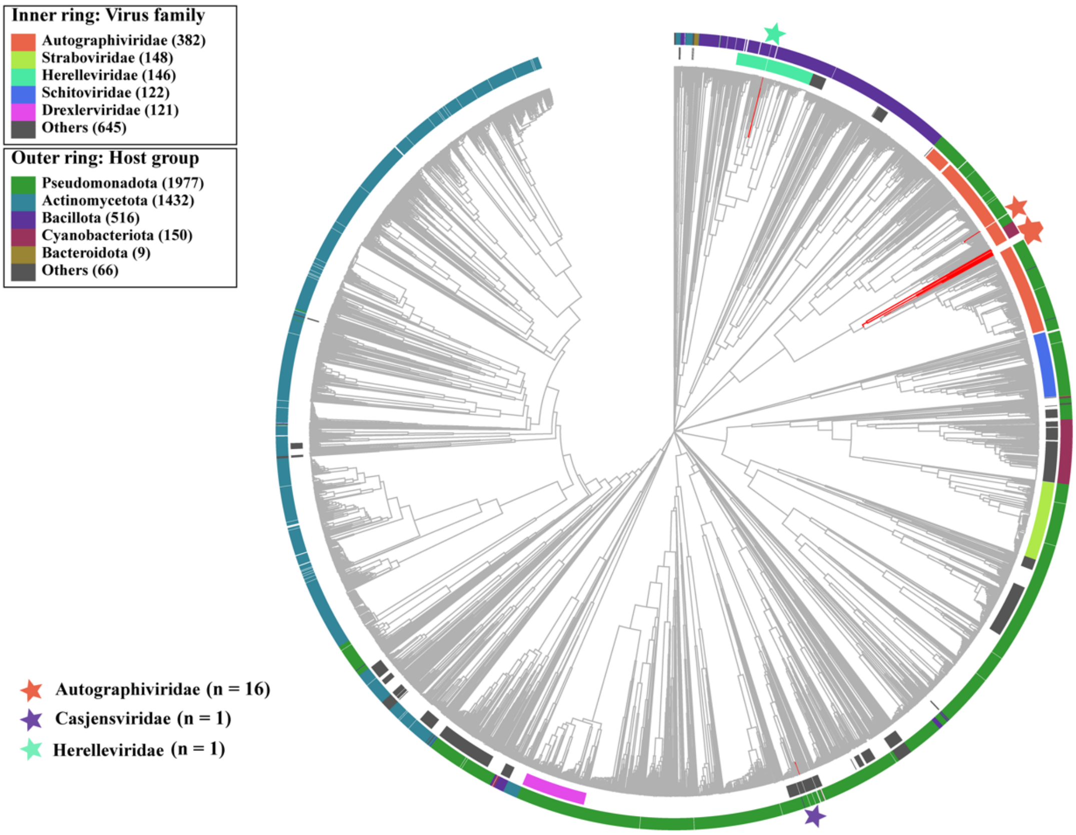

Figure S1 Phylogenetic tree of koala gut viruses and reference viruses based on the TerL gene of *Caudoviricetes*. 18 gut viral genomes are represented, and reference viral genomes are grouped into a tree representing proteome-wide similarity. The phylogenetic tree is midpoint rooted. The inner ring represents the virus family, while the outer ring represents the host group. The red branches and stars represent the koala gut vOTUs. The grey branches represent reference dsDNA viral sequences.

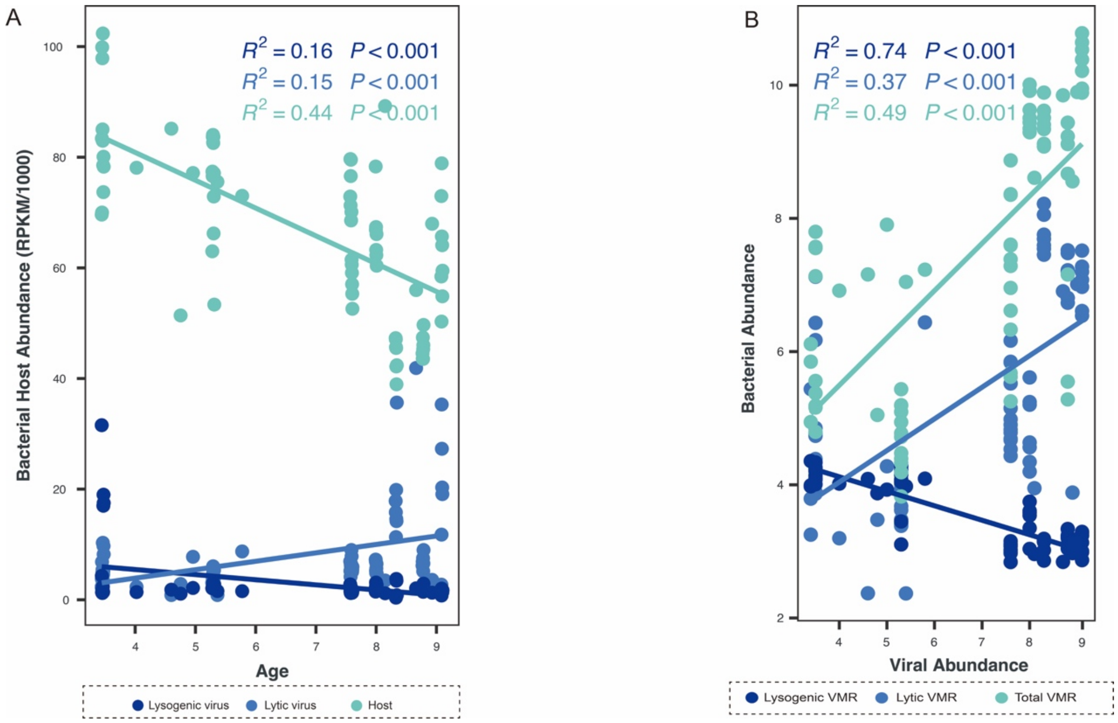

Figure S2 Overview the relationship of koala viruses and their bacterial hosts. (A) Age-dependent pattern of gut lytic and lysogenic virus-host interactions. **(B)** Correlation between age and the relative abundance of virus-to-microbe ratios (VMR).
