# Supplementary material for: Age-dependent patterns of the gut microbiome, antibiotic resistome, and pathogenicity in captive koalas (Phascolarctos cinereus)
Source: Commun Biol. 2025 Dec 7;9:40. doi: 10.1038/s42003-025-09302-2 (PMC12783762; doi:10.1038/s42003-025-09302-2)
Supplement: Supplementary file 2 — Description of Additional Supplementary Files [file 42003_2025_9302_MOESM2_ESM.docx]

1 Description of Additional Supplementary Files

1. **File name:** Supplementary Data 1
2. **Description:** Annotation output of Metagenome-assembled genomes (MAGs) by GTDB-Tk

4

1. **File name:** Supplementary Data 2
2. **Description:** The Information about the mobile genetic elements (MGEs) annotation

7

1. **File name:** Supplementary Data 3
2. **Description:** The Information about the virulence factors (VFs) annotation

10
