# Supplementary material for: Age-dependent patterns of the gut microbiome, antibiotic resistome, and pathogenicity in captive koalas (Phascolarctos cinereus)
Source: Commun Biol. 2025 Dec 7;9:40. doi: 10.1038/s42003-025-09302-2 (PMC12783762; doi:10.1038/s42003-025-09302-2)
Supplement: Supplementary file 6 — Reporting Summary [file 42003_2025_9302_MOESM6_ESM.pdf]

nature portfolio

Corresponding author(s): Jianguo He

Last updated by author(s): Nov 14, 2025

Reporting Summary

Nature Portfolio wishes to improve the reproducibility of the work that we publish. This form provides structure for consistency and transparency in reporting. For further information on Nature Portfolio policies, see our Editorial Policies and the Editorial Policy Checklist.

Statistics

For all statistical analyses, confirm that the following items are present in the figure legend, table legend, main text, or Methods section.

n/a	Confirmed	
☐	☒	The exact sample size (*n*) for each experimental group/condition, given as a discrete number and unit of measurement
☐	☒	A statement on whether measurements were taken from distinct samples or whether the same sample was measured repeatedly
☐	☒	The statistical test(s) used AND whether they are one- or two-sided<br>*Only common tests should be described solely by name; describe more complex techniques in the Methods section.*
☐	☒	A description of all covariates tested
☐	☒	A description of any assumptions or corrections, such as tests of normality and adjustment for multiple comparisons
☐	☒	A full description of the statistical parameters including central tendency (e.g. means) or other basic estimates (e.g. regression coefficient) AND variation (e.g. standard deviation) or associated estimates of uncertainty (e.g. confidence intervals)
☐	☒	For null hypothesis testing, the test statistic (e.g. *F*, *t*, *r*) with confidence intervals, effect sizes, degrees of freedom and *P* value noted<br>*Give P values as exact values whenever suitable.*
☐	☒	For Bayesian analysis, information on the choice of priors and Markov chain Monte Carlo settings
☐	☒	For hierarchical and complex designs, identification of the appropriate level for tests and full reporting of outcomes
☐	☒	Estimates of effect sizes (e.g. Cohen's *d*, Pearson's *r*), indicating how they were calculated

Our web collection on statistics for biologists contains articles on many of the points above.

Software and code

Policy information about availability of computer code

Data collection	Fecal samples were collected from 75 captive koalas housed at Guangzhou Chimelong Safari Park, China. All animals were born and raised in captivity and are descendants of 13 founder individuals originally imported from Currumbin Wildlife Sanctuary, Australia. Koalas were maintained on a standardized diet of fresh eucalyptus leaves (primarily Eucalyptus robusta and Eucalyptus tereticornis). Each animal contributed a single fecal sample, collected at 4 °C and stored at −80 °C until DNA extraction. Samples were stratified by age into three groups: young (1–3 years, n = 12), adult (4–6 years, n = 18), and older (7–9 years, n = 45). DNA was extracted using the QIAamp PowerFecal DNA Kit (QIAGEN, USA), and sequencing libraries were prepared with the NEXTFLEX Rapid DNA-Seq Kit (BioO, USA). Libraries were sequenced on the Illumina NovaSeq 6000 platform using the NovaSeq 6000 S4 Reagent Kit v1.5.
Data analysis	Raw reads were quality-filtered with fastp and assembled using MEGAHIT. MAGs were generated and refined with MetaWRAP and dereplicated by dRep, with taxonomy assigned by GTDB-Tk (GTDB r202). Viral contigs were identified with CheckV and geNomad, clustered with CD-HIT, and taxonomically classified using geNomad and vConTACT3. Viral host prediction was performed using CRISPR spacer matching, tRNA sharing, sequence homology, and iPHoP. Antibiotic resistance genes were annotated with ARG-OAP against the SARG database, while MGEs and virulence factors were identified using BLAST-based searches, PlasFlow, eggNOG-mapper, and METABOLIC. Phylogenetic analyses used MAFFT, trimAl, and IQ-TREE2. Relative abundances were estimated by read mapping with Bowtie2 and normalized using CoverM. Statistical analyses, including alpha- and beta-diversity, NMDS, and PCoA, were performed in R with vegan, microeco, and ggplot2.

For manuscripts utilizing custom algorithms or software that are central to the research but not yet described in published literature, software must be made available to editors and reviewers. We strongly encourage code deposition in a community repository (e.g. GitHub). See the Nature Portfolio guidelines for submitting code & software for further information.

Data

Policy information about availability of data

All manuscripts must include a data availability statement. This statement should provide the following information, where applicable:

- Accession codes, unique identifiers, or web links for publicly available datasets
- A description of any restrictions on data availability
- For clinical datasets or third party data, please ensure that the statement adheres to our policy

All other necessary data supporting the conclusions of this manuscript have been submitted. All sequencing data from this study are available at the China National Center for Bioinformation (https://ngdc.cncb.ac.cn) under the link (https://ngdc.cncb.ac.cn/gsa/s/5Sm94WKE).

Research involving human participants, their data, or biological material

Policy information about studies with human participants or human data. See also policy information about sex, gender (identity/presentation), and sexual orientation and race, ethnicity and racism.

Reporting on sex and gender	This study did not involve human participants, human data, or human biological material
Reporting on race, ethnicity, or other socially relevant groupings	This study did not involve human participants, human data, or human biological material
Population characteristics	This study did not involve human participants, human data, or human biological material
Recruitment	This study did not involve human participants, human data, or human biological material
Ethics oversight	This study did not involve human participants, human data, or human biological material

Note that full information on the approval of the study protocol must also be provided in the manuscript.

Field-specific reporting

Please select the one below that is the best fit for your research. If you are not sure, read the appropriate sections before making your selection.

☒ Life sciences ☐ Behavioural & social sciences ☐ Ecological, evolutionary & environmental sciences

For a reference copy of the document with all sections, see nature.com/documents/nr-reporting-summary-flat.pdf

Life sciences study design

All studies must disclose on these points even when the disclosure is negative.

Sample size	A total of 75 fecal samples were collected from captive koalas, with each animal contributing a single sample. Samples were divided by age group: young (1–3 years, n = 12), adult (4–6 years, n = 18), and older (7–9 years, n = 45).
Data exclusions	Based on reviewer recommendations, seven samples were excluded from the analysis due to possible outlying data points, including four of the youngest and three of the oldest individuals. The remaining 75 fecal samples were included in all downstream analyses
Replication	Note that only a single fecal sample was collected from each animal, representing a snapshot of the gut microbiome at the time of sampling.
Randomization	No randomization was performed, as samples were collected from naturally occurring age groups of captive koalas. All available samples were included according to age categories (young, adult, older).
Blinding	Blinding was not applicable, as all samples were processed and analyzed using automated pipelines without subjective evaluation.

Reporting for specific materials, systems and methods

We require information from authors about some types of materials, experimental systems and methods used in many studies. Here, indicate whether each material, system or method listed is relevant to your study. If you are not sure if a list item applies to your research, read the appropriate section before selecting a response.

Materials & experimental systems

n/a	Involved in the study
☒	☐ Antibodies
☒	☐ Eukaryotic cell lines
☒	☐ Palaeontology and archaeology
☐	☒ Animals and other organisms
☒	☐ Clinical data
☒	☐ Dual use research of concern
☒	☐ Plants

Methods

n/a	Involved in the study
☒	☐ ChIP-seq
☒	☐ Flow cytometry
☒	☐ MRI-based neuroimaging

Animals and other research organisms

Policy information about studies involving animals; ARRIVE guidelines recommended for reporting animal research, and Sex and Gender in Research

Laboratory animals	All laboratory animals in this study were captive koalas housed at Guangzhou Chimelong Safari Park, China. All individuals were born and raised in captivity and are descendants of 13 founder animals originally imported from Currumbin Wildlife Sanctuary, Australia. Koalas were maintained on a standardized diet of fresh eucalyptus leaves (primarily Eucalyptus robusta and Eucalyptus tereticornis) and had no exposure to natural habitats. Samples were stratified by age: young (1–3 years, n = 12), adult (4–6 years, n = 18), and older (7–9 years, n = 45). No experimental interventions were performed, and all fecal samples were collected non-invasively
Wild animals	No wild animals were used in this study. All samples were collected from captive koalas housed at Guangzhou Chimelong Safari Park, China.
Reporting on sex	Sex was not a focus of this study, as all samples were collected from non-human animals and were analyzed without consideration of sex differences.
Field-collected samples	No field-collected samples were used in this study. All fecal samples were collected from captive koalas housed at Guangzhou Chimelong Safari Park, China.
Ethics oversight	All sample collection and experimental procedures were conducted in accordance with the guidelines for the care and use of animals at Guangzhou Chimelong Safari Park. As the study involved only non-invasive collection of fecal samples from captive animals, formal ethics approval was not required.

Note that full information on the approval of the study protocol must also be provided in the manuscript.

Plants

Seed stocks	No plants were directly studied in this research. Although koalas were fed eucalyptus leaves, the study focused on the gut microbiome and did not involve experimental manipulation or analysis of plant material.
Novel plant genotypes	No plants were directly studied in this research. Although koalas were fed eucalyptus leaves, the study focused on the gut microbiome and did not involve experimental manipulation or analysis of plant material.
Authentication	No plants were directly studied in this research. Although koalas were fed eucalyptus leaves, the study focused on the gut microbiome and did not involve experimental manipulation or analysis of plant material.
